# Goal-Focused Admissions for EUPD: An Audit of the NHFT Complex Emotional Needs Inpatient Pathway

**DOI:** 10.1192/bjo.2026.11658

**Published:** 2026-06-30

**Authors:** Sanaa Moledina, Harshani Yapa-Bandara, Marlene Kelbrick

**Affiliations:** 1 Cambridgeshire and Peterborough NHS Foundation Trust, Cambridge, United Kingdom; 2 Northamptonshire Healthcare NHS Foundation Trust, Northampton, United Kingdom

## Abstract

**Aims:**

Prolonged inpatient psychiatric admissions are unlikely to benefit individuals with Emotionally Unstable Personality Disorder (EUPD)/Complex Emotional Needs (CEN) and may even be counterproductive, disrupting daily routines, weakening stabilizing relationships and social roles, reinforcing avoidance of psychosocial stressors, and increasing risk behaviours aimed at maintaining institutional attachments or eliciting care, ultimately impeding recovery.

The Northamptonshire Healthcare NHS Foundation Trust (NHFT) CEN guideline outlines an Inpatient Personality Disorder Pathway enabling ‘Goal Focused Admissions’, requiring pre-admission goals and recommendations, care planning and engagement contracts within 24 hours, consultant review within 72 hours, and brief admissions under 14 days. This audit studied adherence to the CEN guideline.

**Methods:**

Patients with EUPD admitted between 1 January and 28 February 2025 wereincluded; those with comorbid diagnoses were also included if EUPD was the primary reason for admission and remained the primary discharge diagnosis. Referral letters were audited for legal status and short and long-term treatment goals while electronic records were audited for time from admission to documentation of admission goals, engagement contracts, discharge planning, first psychiatrist review, and total length of stay.

**Results:**

Twenty-one patients were included; 81% were informal admissions and the remainder were detained under Section 2 of the Mental Health Act. Crisis resolution was the most frequently cited admission goal (in 48% of cases with documented goals); however, around half of all patients lacked clearly documented admission goals. Admissions were primarily for risk and safety management (42%), followed by medication review (37%), psychological input (16%), multidisciplinary liaison and/or social support (17%), and diagnosis review (5%) were also suggested, with recommendations for referral to other services (CMHT, Personality Disorder Hub etc). Admission goals were documented within 24 hours for 57% of patients, though only 9.5% had engagement contracts completed and 48% had discharge dates recorded within this timeframe. First consultant reviews occurred within the 72-hour target for 48%; delays ranged from 4 to 10 days for the remainder. Only 21% remained inpatient for ≤2 weeks, while 26% of patients stayed 5 weeks or longer.

**Conclusion:**

This audit demonstrated low adherence to the CEN management guideline. Referrals lacked clear admission goals, delaying care planning, engagement contracts, and consultant reviews while extending hospital stays and undermining the collaborative planning and clear therapeutic boundaries fundamental to working with individuals with EUPD. Recommendations include staff training in Structured Clinical Management, group supervision and case discussions with community PD services, and greater guideline awareness among clinicians. Re-audit is advised to monitor improvement.

